# Evaluation of methods for detecting human reads in microbial sequencing datasets

**DOI:** 10.1099/mgen.0.000393

**Published:** 2020-06-19

**Authors:** Stephen J. Bush, Thomas R. Connor, Tim E.A. Peto, Derrick W. Crook, A. Sarah Walker

**Affiliations:** ^1^​ Nuffield Department of Medicine, University of Oxford, Oxford, UK; ^2^​ Organisms and Environment Division, School of Biosciences, Cardiff University, Cardiff, Wales, UK; ^3^​ Public Health Wales, University Hospital of Wales, Cardiff, UK; ^4^​ National Institute for Health Research Health Research Protection Unit in Healthcare Associated Infections and Antimicrobial Resistance at University of Oxford in partnership with Public Health England, Oxford, UK; ^5^​ National Institute for Health Research Oxford Biomedical Research Centre, Oxford, UK

**Keywords:** contamination, human, read depletion, read removal

## Abstract

Sequencing data from host-associated microbes can often be contaminated by the body of the investigator or research subject. Human DNA is typically removed from microbial reads either by subtractive alignment (dropping all reads that map to the human genome) or by using a read classification tool to predict those of human origin, and then discarding them. To inform best practice guidelines, we benchmarked eight alignment-based and two classification-based methods of human read detection using simulated data from 10 clinically prevalent bacteria and three viruses, into which contaminating human reads had been added. While the majority of methods successfully detected >99 % of the human reads, they were distinguishable by variance. The most precise methods, with negligible variance, were Bowtie2 and SNAP, both of which misidentified few, if any, bacterial reads (and no viral reads) as human. While correctly detecting a similar number of human reads, methods based on taxonomic classification, such as Kraken2 and Centrifuge, could misclassify bacterial reads as human, although the extent of this was species-specific. Among the most sensitive methods of human read detection was BWA, although this also made the greatest number of false positive classifications. Across all methods, the set of human reads not identified as such, although often representing <0.1 % of the total reads, were non-randomly distributed along the human genome with many originating from the repeat-rich sex chromosomes. For viral reads and longer (>300 bp) bacterial reads, the highest performing approaches were classification-based, using Kraken2 or Centrifuge. For shorter (c. 150 bp) bacterial reads, combining multiple methods of human read detection maximized the recovery of human reads from contaminated short read datasets without being compromised by false positives. A particularly high-performance approach with shorter bacterial reads was a two-stage classification using Bowtie2 followed by SNAP. Using this approach, we re-examined 11 577 publicly archived bacterial read sets for hitherto undetected human contamination. We were able to extract a sufficient number of reads to call known human SNPs, including those with clinical significance, in 6 % of the samples. These results show that phenotypically distinct human sequence is detectable in publicly archived microbial read datasets.

## Data Summary

All simulations conducted in this study use publicly available third-party software. All data and parameters necessary to replicate these simulations are provided within the article or through supplementary data files. In total, 356 BioProject and >11 000 SRA sample accessions, representing real sequencing data sampled from public archives, are listed in Table S8.

**Impact Statement**

Short-read sequencing data from microbial species can often be contaminated with DNA from the body of the investigator or research subject. It is common practice to remove reads containing human DNA before any further analysis, with two main methods for doing so: mapping all reads to the human genome (and discarding those that do) or using a taxonomic classifier to predict the origin of each read. Despite being a routine task, there are few formal evaluations on the relative performance of these methods. We used simulated data to benchmark a range of tools (for read-mapping and for classification) and found that although many are precise, successfully detecting the vast majority of human reads, reads from certain regions were disproportionately missed. We reasoned that as a consequence there may be hitherto undetected human contamination within publicly archived (and nominally pure) microbial read datasets. Using one of the higher-performing methods identified through simulation, we re-examined >11 000 public datasets for human contamination. In approximately 6 % of these datasets, we could detect enough human reads so as to predict variants – potentially distinguishing characteristics of the human in question. This draws attention to the need for more rigorous approaches of detecting (and removing) human DNA from microbial samples.

## Introduction

Sequencing data from host-associated microbes, including metagenomic read sets, can often be contaminated by the body of the investigator or research subject [1]. Furthermore, as the human genome is around 1000-fold larger than most bacterial genomes, sequencing a tissue biopsy containing equal numbers of human and bacterial cells would still produce a sample containing 99.9 % human DNA [2]. Accordingly, (human) contaminants need to be removed prior to downstream analysis [3] so as to minimize false-positive associations and reduce batch effects. The incomplete removal of human reads from nominally pure microbial read datasets could theoretically lead to large volumes of residual human DNA being deposited in public archives. This raises numerous ethical concerns [4], particularly as individuals can be distinguished even within large, pooled, genomic datasets [5]. This is not just a theoretical problem: previous studies have identified substantial cross-species contamination in genome assemblies [6], including human DNA comprising 2 % of the purportedly complete *Plasmodium gaboni* assembly, a known human parasite [7], and 492 of 2749 non-primate assemblies containing the primate-specific AluY element [8].

With ever-increasing volumes of genomic (and metagenomic) data being deposited in public archives, there is a practical need to benchmark methods of human read detection so as to inform best practice guidelines. These methods follow two basic approaches: subtractive alignment and direct classification. For the former, reads are mapped to the human genome using a short read aligner such as BWA-mem (as in [9, 10]), Bowtie2 (as in [11, 12]) or SNAP (as in [13], or as part of the SURPI – Sequence-based Ultra-Rapid Pathogen Identification – pipeline [14]) with successfully mapped reads then subtracted from the dataset. Numerous pre-processing tools have been developed using this approach including CS-SCORE [15], DeconSeq [16], GenCoF [17] and MetaGeniE [18] (which employ BWA-fastmap, BWA-sw, Bowtie2, and both BWA-mem and Bowtie2, respectively). The second approach is to classify, and then discard, human content by predicting the taxonomic origin of each read using a database of human, bacterial and viral genomes, such as RefSeq or NCBI nr, using k-mer-based classification tools such as Centrifuge [19] or Kraken [20], as in two studies employing the latter [2, 21].

However, no comparisons to date have identified the optimal method. We therefore evaluated several variations on these two basic approaches: by mapping all reads within a mixed dataset to the human genome (using 8 aligners × 2 human genome assemblies), and by predicting read origin with the taxonomic classifiers Centrifuge and Kraken2 [22], using both all-species and human-only databases (2 classifiers × 2 databases). In total, this represents 16 different approaches to subtractive alignment and four different approaches to direct classification, 20 methods in total. To evaluate each method, we simulated 150 and 300 bp paired-end reads at 10-fold coverage from 10 clinically common bacteria used in a previous benchmarking study [23], and at 100-fold coverage from three viral genomes, adding human reads at 1–10 % increments. As well as reporting performance metrics, we characterize those regions of the human genome more likely to be unidentified and which would be inadvertently (and disproportionately) retained in an otherwise ‘human-depleted’ microbial read dataset. We were specifically interested in methods that minimized the number of false negative calls and so also evaluated two-stage approaches, testing the sequential use of different aligners or classifiers. Finally, using one of the highest-performing two-stage approaches, we re-examined 11 577 publicly archived bacterial read sets to identify hitherto undetected human contamination.

## Results and discussion

### Comparing methods for detecting human reads in a contaminated microbial read dataset

**Fig. 1.**
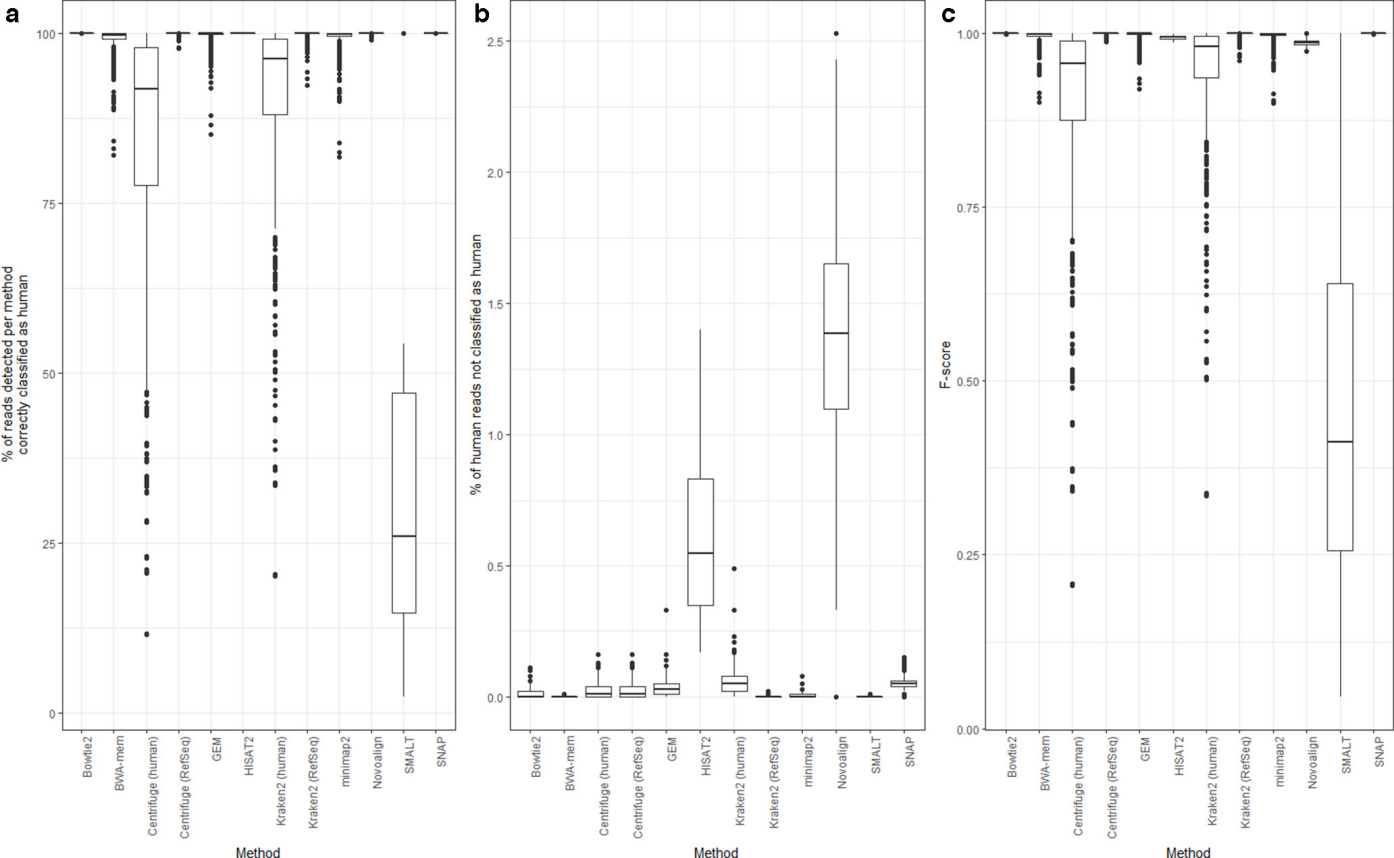
Performance of 12 different methods of identifying human reads within a range of microbial read datasets (comprising 10 bacterial and three viral species sequenced at an average base-level coverage of 10- and 100-fold, respectively, each with between 1 and 10% simulated human contamination, using both 150 and 300 bp reads). All reads were simulated from, and where relevant aligned to, human genome version GRCh38.p12. The subfigures show (a) the percentage of reads per method correctly classified as human, (b) the percentage of human reads not classified as human and (c) the *F*-score. Note that in order to demonstrate the variance between methods in (b), the *y*-axis does not have the same scale as that for (a) and (c). Data for this figure are available in Table S2.

We evaluated the performance of 20 methods of human read detection, comprising two read classifiers (Centrifuge and Kraken2), both used with two different databases, and eight aligners (Bowtie2 [24], BWA-mem [25], GEM [26], HISAT2 [27], minimap2 [28], Novoalign (www.novocraft.com), SMALT (http://www.sanger.ac.uk/science/tools/smalt-0) and SNAP [29]), each aligning reads to two different versions of the human primary assembly, GRCh38 and GRCh37 (the latter of lower quality). Each method was evaluated using reads simulated from 10 closed bacterial genomes (the Gram-positive *Clostridioides difficile *, *Listeria monocytogenes *, *Staphylococcus aureus* and *Streptococcus pneumoniae *, the Gram-negative *Escherichia coli *, *Klebsiella pneumoniae, Neisseria gonorrhoeae*, *Salmonella enterica* and *Shigella dysenteriae *, and *Mycobacterium tuberculosis *) and three viral genomes [hepatitis C, human immunodeficiency virus (HIV), influenza A] (Table S1, available in the online version of this article), to which were added reads simulated from human genome GRCh38. We included the lower quality (and hence less accurate/complete) assembly in the approaches tested to demonstrate how the detection of human contamination differs between the two versions. This comparison contained 9360 records, comprising two read lengths (150 and 300 bp, characteristic of the Illumina NextSeq and MiSeq platforms, respectively) × 3 replicates × 13 species × 10 incremental additions of human reads (comprising 1–10 % of the total number of bacterial or viral reads) × (8 aligners+4 classifier/database pairs), for a total of 780 records per aligner/classifier+database, of which 600 were bacterial. The number of reads simulated and the performance statistics for each method – the percentage of reads correctly classified as human (‘true positive rate’), the percentage of human reads not classified as human (‘false negative rate’), positive predictive value (the proportion of reads classified as human that truly are human), recall (sensitivity) and the F-score (harmonic mean of precision and recall) [30] – are given in Table S2, with distributions illustrated in Fig. 1a.

Although the median percentage of true positive classifications was high for the majority of methods, they were distinguishable by variance. This was only the case for bacteria as all methods using viral genomes identified 100 % of the human reads (Table S2). With bacteria, the highest true positive rates (>99.9 %), with negligible variance, were found using Bowtie2, HISAT2 or SNAP when aligning reads against the human genome. The true positive rate was 100 % in 582 of the 600 (bacterial) simulations using SNAP, 594 of the 600 simulations using Bowtie2, and all 600 simulations using HISAT2 (Table S2). Similarly high true positive rates (around 99 %) were found when predicting human reads using a taxonomic classifier (Centrifuge/Kraken2) with the RefSeq database (i.e. the human genome plus microbial genomes) and when using Novoalign, BWA-mem, GEM or Minimap2 to align all reads against the human genome, although in each case the variance in *F*-score was far higher compared to Bowtie2, HISAT2 or SNAP. True positive rates were notably reduced (to <90 %), and variation substantially increased, when using either taxonomic classifier with a human-only database (Fig. 1). The poorest performing method (true positive rate <25 %) was the aligner SMALT which, although correctly identifying all human reads (i.e. having a false negative rate of 0), could not reliably discriminate bacterial from human sequence and so made a large number of false positive calls (Table S2).

**Fig. 2.**
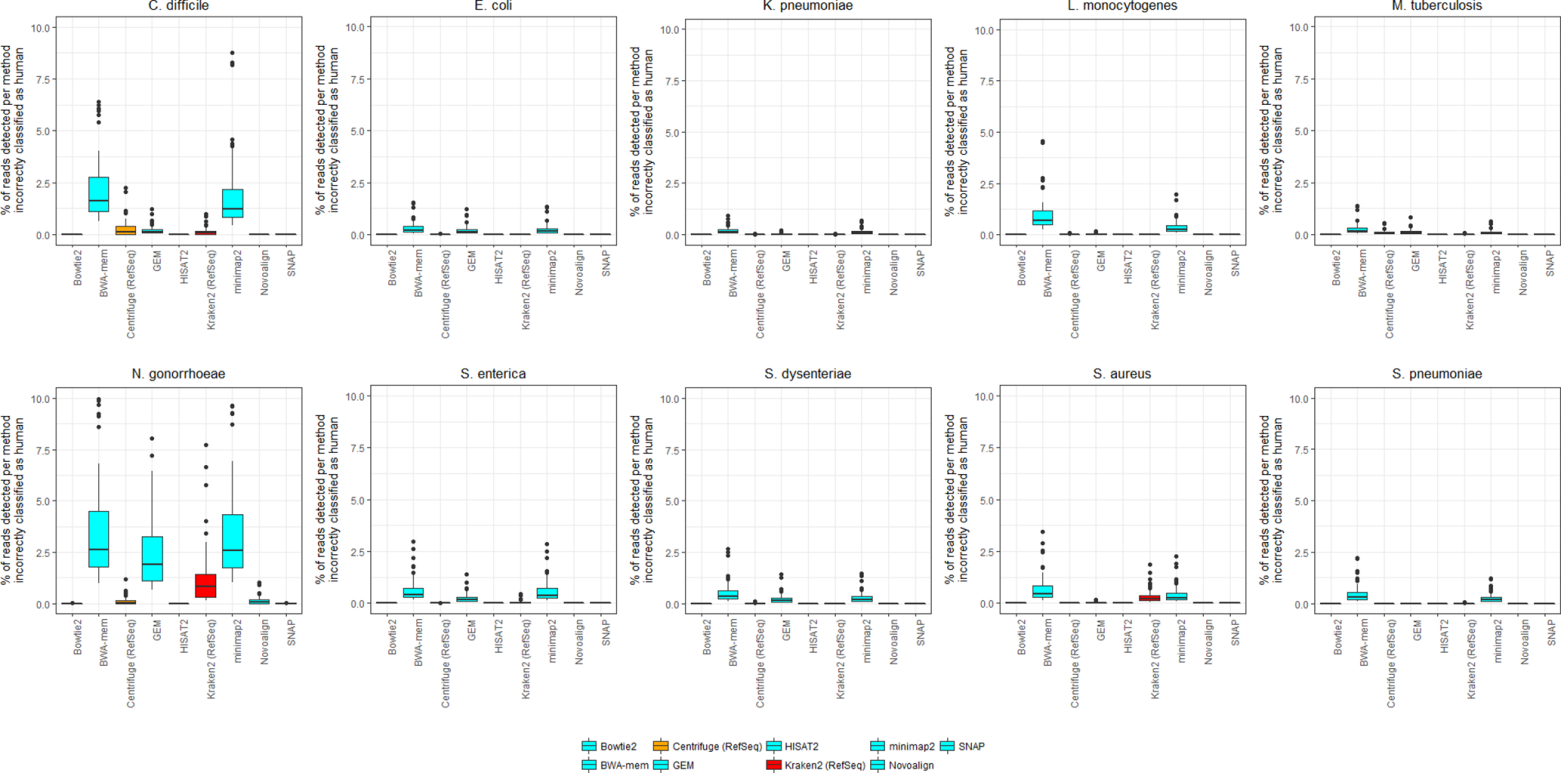
The percentage of reads incorrectly classified as human by nine different methods of human read detection within a range of microbial read datasets, partitioned by species. Data for this figure are available in Table S2 and constitute simulated reads at 10-fold coverage from each of 10 species supplemented with 0–10% human contamination, using both 150 and 300 bp reads. Data from three methods (the aligner SMALT and the classifiers Kraken2 and Centrifuge, each using a human-only database) are not shown. This is because these methods have a very high false positive rate across all species (Fig. 1). Data from viral datasets are not shown because in our simulations no viral read was incorrectly classified as human, by any method (Table S3).

The proportion of false positive calls – while generally low – notably varied by species (Fig. 2). This was particularly apparent for *C. difficile* (the genome of which comprises approx. 11 % mobile genetic elements [31]) and *N. gonorrhoeae* (in which horizontal gene transfer from a human host has previously been characterized [32]) where the aligners BWA-mem and minimap2, and to a lesser extent GEM, made false positive calls at a rate >5 % in some simulations. Importantly, these methods misidentified bacterial reads as human even when no human reads were present in the dataset (Table S2).

Most methods produced a low number of false negative calls, of approximately <1 % of the total reads, with minimal variance (Fig. 1). This was particularly apparent for BWA-mem and Kraken2 (using the RefSeq database), for which the false negative rate using bacterial data was 0 % for all 600 simulations, and for 573 of 600 simulations, respectively, with similar results seen with viral data (Table S2). However, two aligners, HISAT2 and Novoalign, were prominent outliers, having relatively high false negative rates (with bacterial data, median 1.6 and 1.4 %, respectively) and notably greater variance. While HISAT2 had the highest true positive and lowest false positive rate (100 and 0 %, respectively), it also made the greatest number of false negative calls (Fig. 1). A lower number of false negative calls were made by, for example, BWA-mem, although at the expense of a higher false positive call rate. By contrast, Bowtie2 and SNAP had true positive and false positive rates comparable to HISAT2 (i.e. approximately 100 %), although far lower false negative rates.

Unlike the other aligners, the variance in the false negative rate for Bowtie2 was related to read length. For 291 of the 300 bacterial simulations using 150 bp reads, it was notable that the false negative rate was 0 % (Table S2). However, and in clear contrast, for 288 of the 300 simulations using 300 bp reads, the false negative rate was >0 % (median 0.02 %). The differences in false negative rates can be attributed to our usage of default parameters for each aligner. It is important to note that Bowtie2 was originally designed to align shorter reads (<300 bp) with high sensitivity, with its scoring function optimized to that end. Without modifying the default parameters, longer reads would be slightly less likely to align, consistent with our observations.

**Fig. 3.**
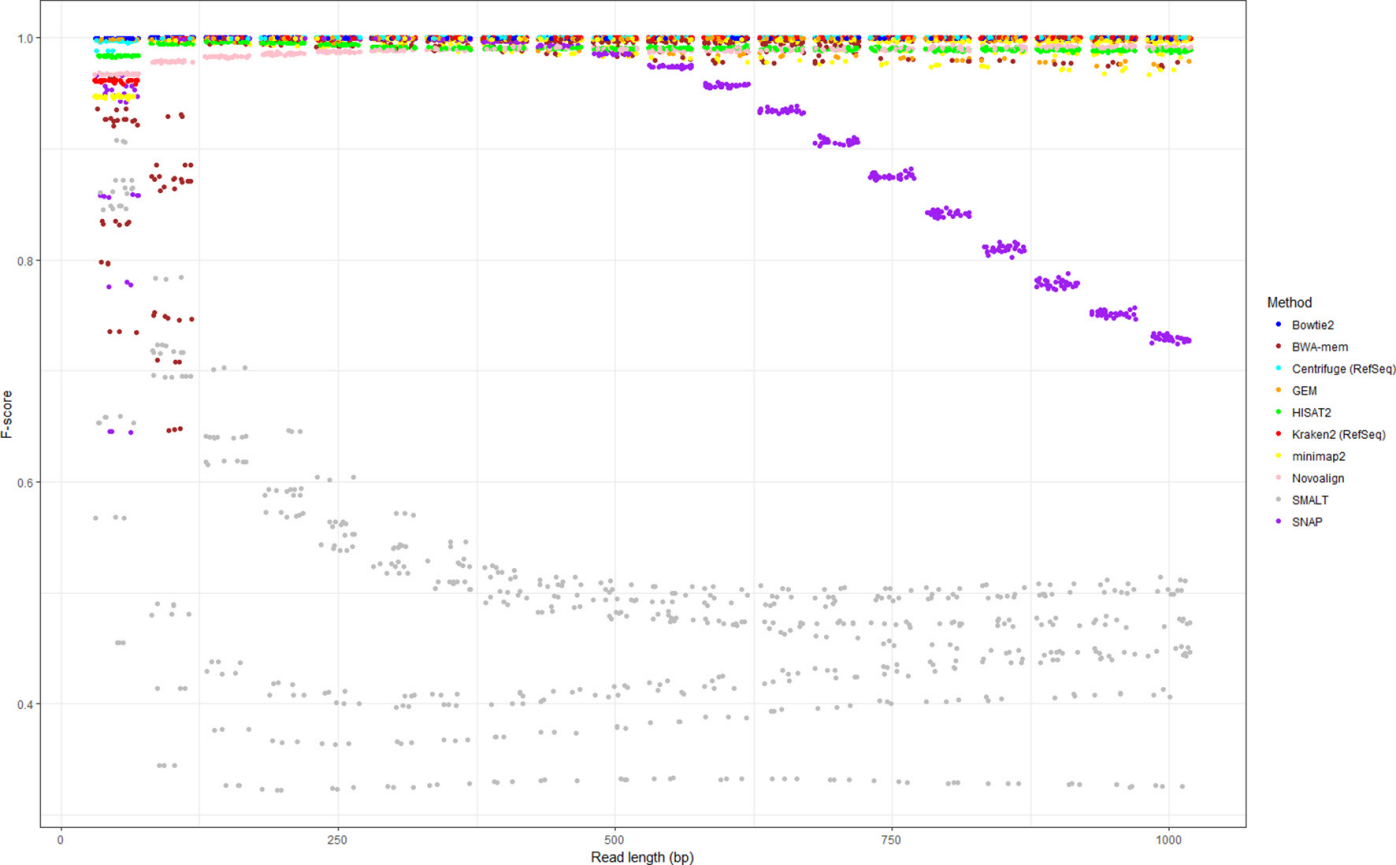
Performance of 10 different methods of identifying human reads within a range of microbial read datasets (comprising 10 bacterial species sequenced at an average base-level coverage of 10-fold, each with 10 % simulated human contamination). All reads were simulated from, and where relevant aligned to, human genome version GRCh38.p12. Each point represents a simulation replicate, coloured according to method. Points are jittered to allow over-plotting. There is considerable overlap between points as many methods perform equivalently highly when using long reads. Data for this figure are available in Table S3.

To test the effect of read length on human read classification, we repeated the above simulations varying read length from 50 to 1000 bp, at 50 bp increments (using the same parameters and formula for insert size as described in the Materials and Methods). We repeated the simulations for the set of 10 bacterial genomes, although we restricted the analysis to 10 methods (we excluded the use of Centrifuge and Kraken2 with human-only databases) and simulated test sets comprising 10 bacterial read sets each with 10 % human contamination. In total, this represents 6000 simulations (20 read lengths × 3 replicates × 10 species × 10 methods). Performance statistics are given in Table S3, and the variance of *F*-score with read length illustrated in Fig. 3. We excluded viral genomes from this analysis as the previous simulations showed that viral reads could be unambiguously distinguished from human reads.

For longer (>300 bp) bacterial reads, the classification-based methods (Centrifuge and Kraken2) consistently produced precision, recall and *F*-scores of 1 (Table S3). By contrast, alignment-based methods were especially sensitive to read length because implicitly this affects the appropriateness of the parameters used to score alignments (the default parameters of most aligners assume reads of shorter length). All methods performed with comparable *F*-scores if using reads within the range of 150–300 bp (i.e. read lengths expected of Illumina HiSeq/NextSeq/MiSeq sequencers), although there was considerable variance in performance at lower read lengths, with the exception of Bowtie2 (which performed well even with 50 bp reads). SNAP declined in performance sharply when using reads greater than 400 bp, although doing so is contrary to recommended use (to enable SNAP to process reads >400 bp, the value of MAX_READ_LENGTH needed to be manually edited in SNAPLib/Read.h before compiling). SMALT, if used with default parameters, appeared optimized for shorter (<250 bp) reads, and showed consistently poorer performance (plateauing at an approximate *F*-score of 0.5) for longer reads. SMALT was also strongly affected by species (see the highly variable *F*-score in Fig. 1c), with multiple *F*-score distributions apparent in Fig. 3.

We found no discernible difference in true or false positive classification rates when aligning reads to lower-quality human genomes (i.e. older assemblies) (Fig. S1).

### All methods of human read depletion fail to classify reads from similar regions

We were particularly interested in false negative calls – human reads that were not classified as such – as these could be retained within a microbial read dataset. By reference to the GRCh38.p12 and GRCh37.p13 gene coordinates (from Ensembl BioMart [33]), we identified, per method, the proportion of reads with false negative classifications (i.e. human reads not classified as human; hereafter ‘false negative reads’) deriving from different genomic regions (Table S4). With Kraken2 and Centrifuge (when provided a combined microbial and human database), it was particularly notable that in absolute terms there were not only few false negative calls but few in genic regions (Table S4). This is probably because both methods avoid (intrinsically inexact) alignments to make exact-match queries against a database of *k*-mers, with larger databases – and the uniqueness of gene sequences – affording greater resolving power. While Kraken2 classifies reads on the basis of the lowest common ancestor for all genomes containing its constituent *k*-mers [20], Centrifuge can also assign a single sequence to multiple taxonomic categories. However, as the present task is essentially one of binary classification – discriminating human from non-human reads – we found only a modest difference in performance between the two approaches. Using the RefSeq database, Kraken2 had greater variance in false positive calls than Centrifuge although compensated with fewer false negative calls (Fig. 1). For both methods the presence of at least one or more unique *k*-mers among the reads sequenced from human genes appears sufficient to discriminate them from essentially all bacterial genomes (unique *k*-mers ostensibly reflecting divergent evolutionary histories).

Table 1 summarizes, for each method, the number of unique human genes from which one or more false negative reads were derived, or to which one or more bacterial reads had been classified, across all 13 species and all replicates of each method. Consistent with Fig. 1c, virtually all human reads were detected by methods with zero or negligible false negative rate such as BWA-mem and SMALT, with reads undetected by Kraken2 or Centrifuge (when using the RefSeq database) representing <40 genes. The number of genes identified by the two methods with the highest false negative (HISAT2) and false positive (SMALT) rates accounted for the majority of genes in the human genome (>10 000), suggesting that reads misidentified by these methods were more likely to be randomly distributed across the genome. Accordingly, we would not expect these sets of genes to be significantly enriched for Gene Ontology (GO) terms. However, we found that for the remaining methods (which were more sensitive and/or more precise) each set of genes from which one or more false negative reads were derived was enriched for a broadly consistent set of GO terms related to the nervous system (Table S5). For example, when aligning reads to the GRCh38.p12 assembly, undetected reads were enriched for genes with process terms such as ‘dendrite development’ (among the set of genes with reads undetected by GEM) and ‘glutamatergic synaptic transmission’ (minimap2) and component terms such as ‘synaptic membrane’ (SNAP, GEM and minimap2), ‘postsynaptic membrane’ (Bowtie2), ‘neuromuscular junction’ (GEM), ‘neuron part’ (minimap2), ‘photoreceptor disc membrane’ (Kraken2) and ‘astrocyte projection’ (Centrifuge). Similar results were found when aligning reads to the GRCh37.p13 assembly, with undetected reads enriched for genes with process terms including ‘neural tube formation’ (Bowtie2) and ‘synapse assembly’ (both minimap2 and SNAP) (Table S5). An equivalent set of nervous system-related GO terms was found for each set of genes to which one or more microbial reads had been identified (i.e. genes with a probable microbial homologue) in both assemblies. In this case, misidentified microbial reads were also enriched for genes with component terms such as ‘neuronal cell body’ (GEM; GRCh38.p12) and process terms such as ‘postsynaptic membrane assembly’ (BWA-mem; GRCh37.p13) (Table S6).

**Table 1.** Total number of genes, for each method, from which one or more reads could not be classified as human (i.e. false negative calls) or to which one or more microbial reads had been misclassified (i.e. false positive calls). Note that as the current Centrifuge and Kraken RefSeq-based databases contain human assembly GRCh38, it is unnecessary to create an equivalent with GRCh37

Method	No. of human genes for which one or more reads could not be classified as human, across all species and all replicates of that method (i.e. false negative classifications)		No. of human genes to which one or more microbial reads had been misclassified, across all species and all replicates of that method (i.e. false positive classifications)	
					GRCh38.p12	GRCh37.p13	GRCh38.p12	GRCh37.p13
Bowtie2	627	593	0	0
BWA-mem	0	0	13 170	587
Centrifuge (human)	23	n/a	0	n/a
Centrifuge (RefSeq)	25	n/a	0	n/a
GEM	42	64	120	110
HISAT2	14 943	14 211	0	0
Kraken2 (human)	1547	n/a	0	n/a
Kraken2 (RefSeq)	37	n/a	0	n/a
Novoalign	3588	3367	11	10
minimap2	660	516	443	421
SMALT	0	2	35 518	33 874
SNAP	3283	3047	0	0

**Fig. 4.**
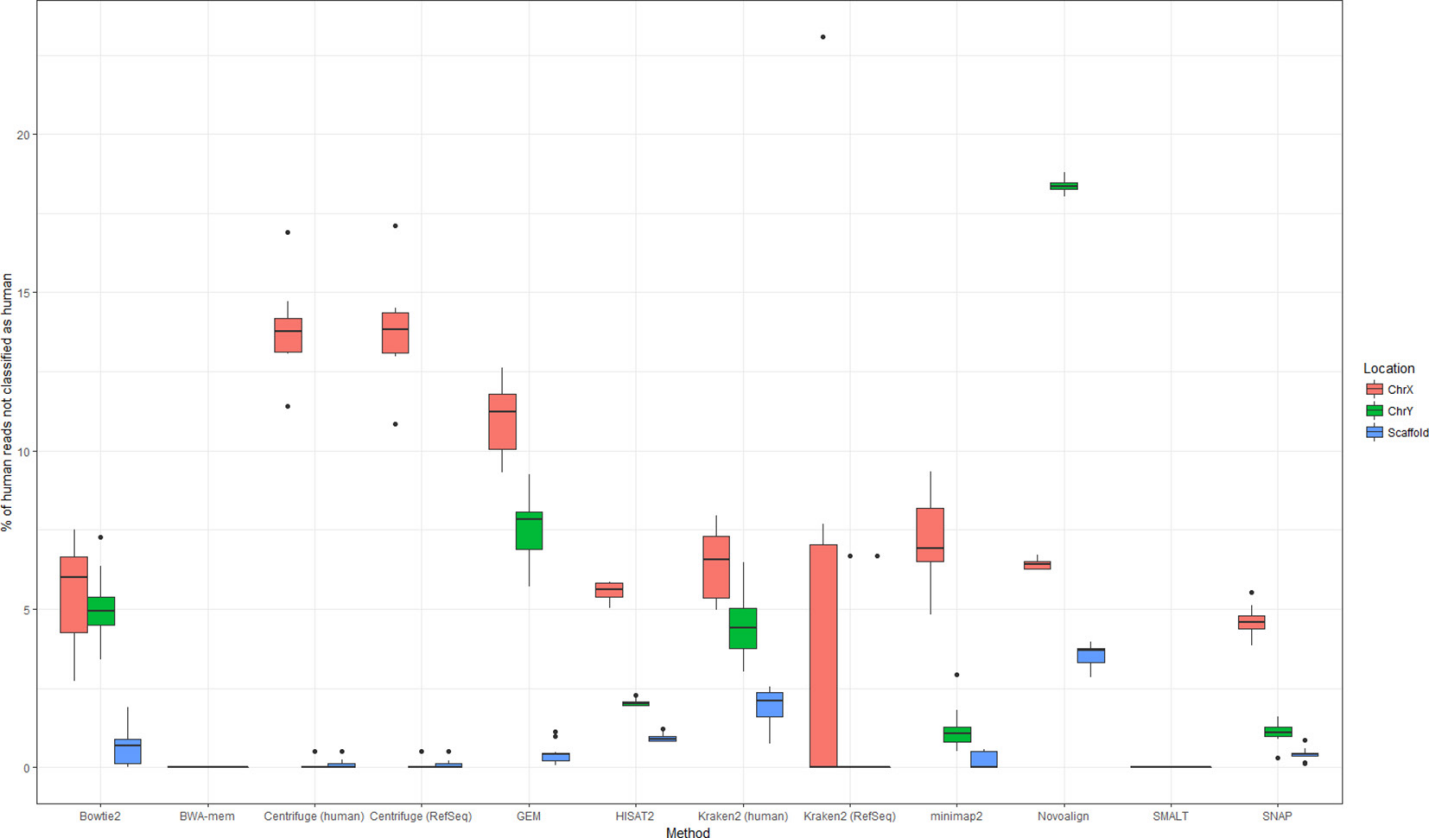
Proportion of human reads not classified as human by nine different methods of human read detection, and their genomic location. Data for this figure are available in Table S4.

These results suggest that, in general, alignment-based methods of human read detection consistently fail to classify reads from functionally similar genes. Further to this, a relatively high proportion of false negative reads originated from the sex chromosomes (Fig. 4, Table S4), both known for their high (>50 %) repeat content [34, 35]. This was particularly apparent when using Novoalign (from which the median percentages of false negative reads from chromosomes X and Y were 6.4 and 18.3 %, respectively), and to a lesser extent Bowtie2 (6.0 % X, 4.9 % Y) and Centrifuge, when using the RefSeq database (13.8 % X, 0 % Y). While it is common practice to repeat-mask genomes prior to alignment [36] (or prior to inclusion in a Centrifuge/Kraken2 *k*-mer database), for the purpose of human read detection, this would probably exacerbate the problem – repetitive or low-complexity reads would not be matched to a masked region.

Overall, the non-random distribution of false negative reads suggests that many methods of human read detection failed to detect reads from similar (repetitive) regions. It follows that a possible strategy for maximizing the number of correctly identified human reads is to combine approaches so that multiple methods could compensate for locally poor mapping.

### Human (short) read detection is improved by combining results from multiple aligners or classifiers

**Fig. 5.**
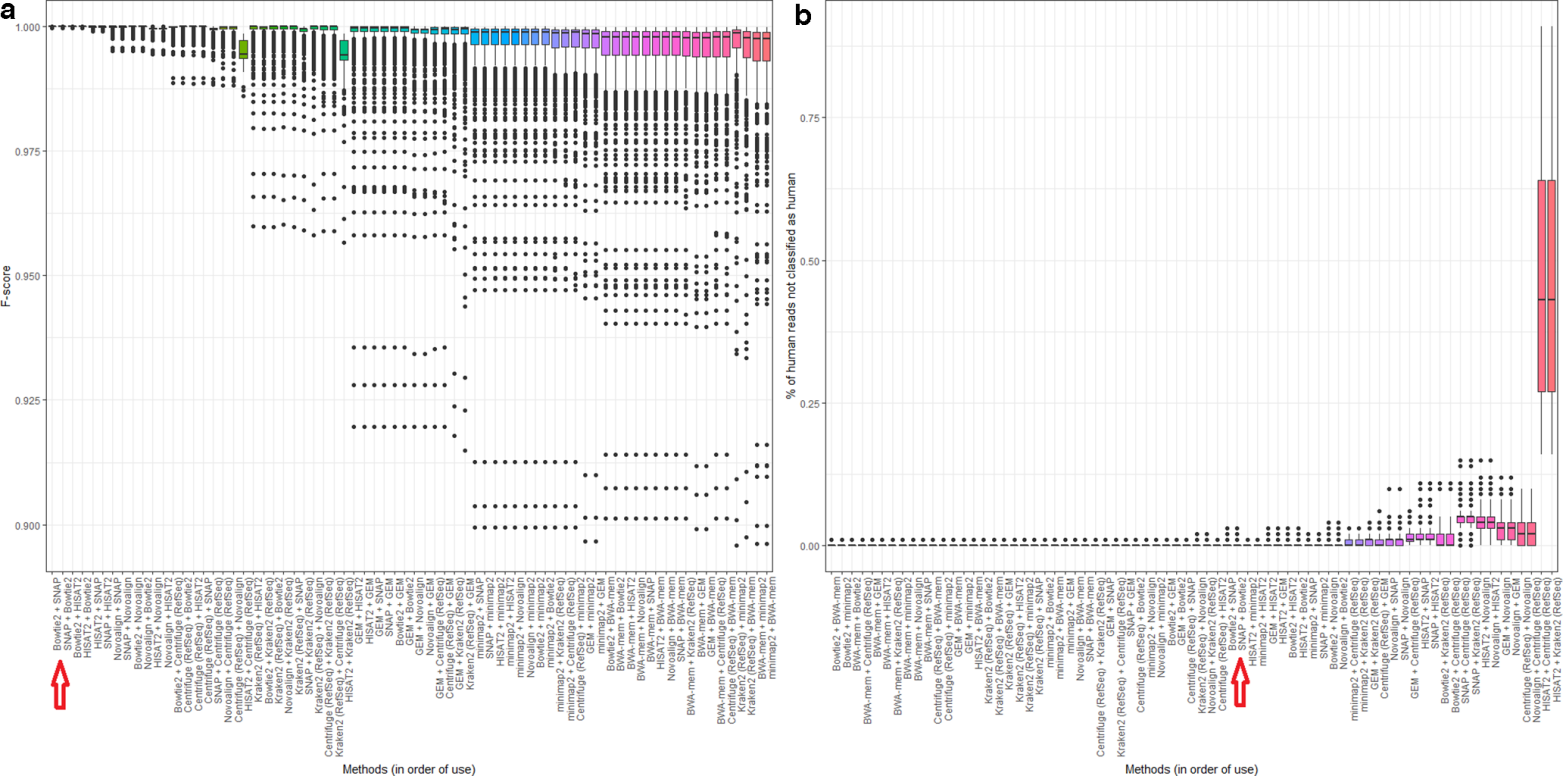
Performance of all two-stage combinations of nine independent methods of identifying human reads within a range of microbial read datasets (72 pairwise combinations; the data comprise 10 bacterial species, each with between 1 and 10% simulated human contamination, using both 150 and 300 bp reads). All reads were simulated from, and where relevant aligned to, human genome version GRCh38.p12. The subfigures show (a) the *F*-score, and (b) the percentage of human reads not classified as human. Bars in both subfigures are ordered from left to right by increasing variance, and in alphabetical order for methods with equal variance. Bowtie2 + SNAP is indicated on the axis. Data for this figure are available in Table S7.

The retention of false negative reads – human reads not identified as such – within a nominally ‘pure’ microbial read dataset is an ethical and practical concern. We reasoned that for contaminated short (150–300 bp) microbial read sets, for which human reads are not consistently removed (Table S2), a two-stage approach – the sequential use of different aligners/classifiers – could map a greater number of reads to the human genome without losing specificity. Accordingly, we re-calculated true positive, false positive and false negative rates after combining the set of classifications made by any two methods. This two-stage approach first classified, and then discarded, ‘human’ reads using one method, and then performed a second round of classification using a second method. In this way, a method with high precision could be supplemented by a method with high sensitivity, maximizing the utility of both. For this analysis we excluded three approaches with high positive rates (Fig. 1) that were unlikely to provide much added-value (SMALT, and human-only databases with Centrifuge and Kraken2). We also restricted this analysis only to bacterial data because our simulations showed that individual methods could discriminate viral from human reads (Table S2). The numbers of reads classified by each pairwise combination of methods are given in Table S7, with *F*-score and false negative rate distributions illustrated in Fig. 5.

While these results broadly recapitulate those of Fig. 1, variance in the false negative rate was substantially reduced when combining methods, suggesting that different aligners could compensate for each other’s omissions. This was particularly apparent when combining the two individually poorest-performing aligners in terms of false negative rates in Fig. 1b, Novoalign and HISAT, which reduced the median false negative rate of each aligner from approximately 1.5 to 0.18 % (Fig. 5) (although these were still by far the worst performing methods for false negative rates, having little utility as the first method in a two-stage process).

The optimal combination of methods, in terms of *F*-score, was Bowtie2 followed by either SNAP or HISAT2. The *F*-score was 1 for 541 of the 600 Bowtie2/SNAP simulations and 416 of the 600 Bowtie2/HISAT2 simulations. Similarly, the false negative rate was 100 % for 588 of the 600 Bowtie2/SNAP simulations and for 471 of the Bowtie2/HISAT2 simulations, although in both cases the absolute number of false negative calls, if any, was extremely low (<10 reads; Table S7).

### Does it matter if only most, but not all, human reads are removed?

In our simulations, relatively few human reads were retained in absolute terms. However, our simulations used relatively low sequencing depths of <800 000 total reads for each of the 10 species (hence, <80 000 true human reads in any one sample, as detailed in Table S1). Although we found that all methods of human read depletion resulted in a consistently small proportion of retained human reads (typically <0.1 %), as sequencing depths increase (concomitant with decreasing cost) this could still be hundreds of human reads in absolute terms. Furthermore, these reads are unlikely to be randomly distributed throughout the human genome, as demonstrated above for the (repetitive) sex chromosomes. Other regions of the human genome may also have similar problems (although we did not directly observe this in our simulations). For instance, the human leukocyte antigen (HLA) on chromosome 6p21.3 is hypervariable, having >10 % sequence divergence between haplotypes [37]. If using a ‘general purpose’ aligner, reads are highly unlikely to map to this region. As such, these reads would be disproportionately retained within a ‘human depleted’ microbial read dataset.

Assuming a sufficient number of reads across a given region, it is possible that variants within it could be called with confidence and used to impute phenotype (e.g. variants in HLA genes have well-documented associations with immune disorders [38]) and/or be associated with named individuals, assuming the identities of those involved in sample preparation were also known. Although anonymizing samples is routine practice (and to some extent ameliorates this risk), generators of raw sequencing data frequently waive their own anonymity by virtue of publication. Furthermore, while human variant calls are often filtered on the basis of minimum depth (number of reads covering that position) and base call frequency (proportion of reads supporting a particular allele), in absolute terms this rarely represents large numbers of reads. For instance, the default recommendations applied by the variant calling pipeline NASP [39] are a minimum depth of 10 reads, of which nine must support a given SNP.

To demonstrate that these are not just theoretical concerns, we reasoned that the highest-performing method, the Bowtie2/SNAP two-stage approach, would be able to best recover human reads inadvertently retained within public datasets. To test this possibility, we parsed the European Nucleotide Archive to obtain a diverse range of Illumina paired-end genome sequencing reads from each of the 10 bacterial species used in our simulations (see Materials and Methods). In total, we obtained 11 577 SRA sample IDs, representing sequencing data from 356 different BioProjects. From each sample we identified human reads using the two-stage method of Bowtie2/SNAP and then called SNPs – which could be used to predict individual phenotypic characteristics – using a BWA-mem/mpileup pipeline (see Materials and Methods). The results are summarized in Table 2, with the number of human reads detected per sample, and associated phenotypic predictions, given in Table S8.

**Table 2.** SNPs called from contaminating human reads within publicly archived bacterial sequencing datasets

Status of sample	No. of samples	% of samples
Contains one or more human SNPs	7389	63.8
Contains one or more human SNPs with ≥2 reads supporting alternative allele, QUAL ≥ 20, and MAPQ = 60	1657	14.3
Contains one or more human SNPs considered ‘common’ in dbSNP	5113	44.1
Contains one or more ‘common’ human SNPs, each with ≥2 reads supporting alternative allele, QUAL ≥ 20, and MAPQ = 60	731	6.3
Contains one or more human SNPs recorded in ClinVar	412	3.6
Contains one or more human SNPs recorded in ClinVar, each with ≥2 reads supporting alternative allele, QUAL ≥ 20, and MAPQ = 60	191	1.6
Contains one or more ‘common’ human SNPs recorded in ClinVar, each with ≥2 reads supporting alt allele, QUAL ≥ 20, and MAPQ = 60	13	0.11

While in absolute terms the number of human reads identified in the 11 577 samples was often low, there were several prominent outliers, with 58 samples (0.5 %) containing >10 000 human reads and 299 (2.6 %) containing >1000. Overall, 100 or fewer human reads could be detected in 8321 samples (71.8 %), 10 or fewer human reads in 2550 (22.0 %) and no human reads in 464 (4.0 %). Across the 11 113 samples in which one or more human reads were detected, the mean number of reads detected was 1255. With each sample sequenced at a depth of 1–5 million reads, this represents 0.02–0.1 % of the number originally sequenced, consistent with our expectation from simulations.

It is possible to call SNPs even from a limited number of human reads (and so impute phenotype), although we do not expect the majority of calls to be made with high confidence. Without applying any filter criteria, one or more human SNPs could be called from the residual human reads in 7389 (63.8 %) of samples (Table 2), although it is reasonable to believe a high error rate. We applied several post-processing criteria to retain only higher-confidence SNPs, requiring each alternative allele to be supported by two or more uniquely mapped reads and to have been previously reported in a major human population (i.e. the SNP is considered ‘common’; see Materials and Methods). After applying these criteria, SNPs could still be called in 731 samples (6.3 %) (Table 2).

From these higher-confidence SNPs, it was possible to impute particular phenotypes. For instance, in 13 samples (0.1 %), one or more ‘common’ SNPs were called that were also recorded in the ClinVar database [40]. While these SNPs were all considered ‘benign’ or ‘likely benign’ (Table S8), this is probably because SNPs with pathological clinical significance are uncommon (i.e. this subset of ClinVar SNPs would not meet the dbSNP definition of ‘common’, which is based on minor allele frequency; see Materials and Methods). By relaxing this requirement, we found 191 samples (1.6 %) with one or more SNPs supported by multiple uniquely mapped reads and which were present in ClinVar but not also ‘common’ (Table 2). Within this subset, we found three samples with SNPs indicative of adverse drug responses (ClinVar variant IDs 12351, 17503 and 37340) and 19 samples with pathological SNPs, including hereditary predisposition to various cancers (including breast and ovarian), polycystic kidney disease and Leber’s optic atrophy (Table S8).

These analyses of real data are broad in scope and intended to illustrate a general point: that phenotypes can be imputed from even a small number of reads. Overall, these results establish that phenotypically distinct human sequence is detectable in publicly archived microbial read datasets.

While many human reads will lack phenotypically associated SNPs, the presence of even a small number with them could still allow the possibility of identifying the human source. To eliminate this possibility, it is prudent to take as exhaustive an approach to their removal as practically possible. Nevertheless, as only a proportion of reads will contain SNPs, it is also tempting to apply a reasonably broad level of tolerance to the number of human reads retained within a real bacterial sample. For practical purposes, the optimal trade-off between the two is a function of the time and computational resources available.

**Fig. 6.**
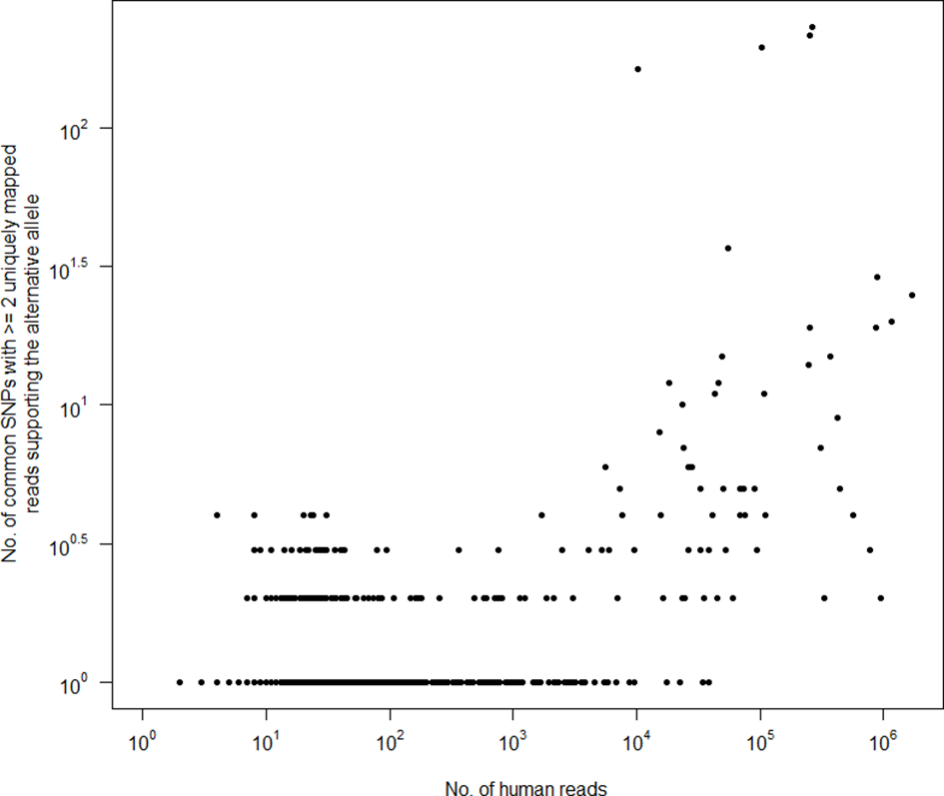
Relationship between the number of human reads retained within 11 577 publicly archived bacterial read sets and the number of higher-confidence ‘common’ SNPs called using them (i.e. SNPs previously called by the 1000 Genomes Project in at least one of 26 major human populations, with the alternative allele supported by ≥2 uniquely mapped reads). Human reads were identified by aligning all reads to the human genome using Bowtie2 followed by SNAP. Data for this figure are available in Table S8.

Across all 11 577 samples, we made 12 215 higher-confidence SNP calls (those where the alternative allele was supported by two or more uniquely mapped reads), of which 2063 were also ‘common’ (Table S8), i.e. more likely to be annotated with phenotypic characteristics. We found that on a per-sample basis, fewer than 10 ‘common’ SNPs could be called with reasonable confidence from samples containing up to 10 000 human reads (Fig. 6). While this suggests a relatively large number of human reads could be retained in a microbial read dataset without being unduly informative, it is important to note that individual SNPs (which could still be clinically significant) could also be called with confidence even at far lower depths (<100 reads; see Fig. 6). Furthermore, there is already a substantial body of overt phenotypic associations with single SNPs, including, for instance, blue eyes [41], lactose intolerance [42] and alcohol-related flushing [43].

### Recommendations for depleting human reads from microbial read datasets

If using shorter (c. 150 bp) bacterial reads, then to maximize the number of human reads whilst minimising the risk of false positive calls, we recommend using Bowtie2 (which performs near-exact read mapping, optimizing parameters where appropriate to account for read length), followed by SNAP. Our analyses suggest that these aligners could complement each other and so the cost of using two methods sequentially would be incurred only in additional computational time, not in reduced accuracy. The individual methods with lowest false negative rates (i.e. those which successfully detected the majority of human reads) were BWA-mem and Kraken2, although these methods both had relatively high false positive rates and so could erroneously classify bacterial reads as human (noting that these false positive rates are relatively high only compared to other methods and that as a proportion of the number of input reads, the number of false positive calls was comparatively low). False positive calls were particularly apparent for genomes rich in mobile elements, such as *C. difficile *.

Classification accuracy was affected by read length, however, particularly when using alignment-based methods (Fig. 3). We found that when sequencing viral reads or bacterial reads of >300 bp, the most accurate, as well as simplest and quickest, approach was to use direct classification methods (Centrifuge and Kraken2). For reproducibility, we used publicly available resources in this study wherever possible, rather than building custom Centrifuge/Kraken2 databases. In the case of Centrifuge, this database was relatively old (June 2016), although because it performed well across multiple metrics, we anticipate that a more up-to-date database would only improve performance further. In addition, although runtime was not formally assessed in this study, we noted that classification-based methods were far faster to complete than alignment-based human read detection. Importantly, and irrespective of the speed of a given aligner, ‘alignment’ is in practice a multi-step process, requiring the subsidiary steps of BAM sorting, BAM indexing, BAM subsetting and read ID extraction (i.e. converting BAM to BED). This approach is intrinsically slower than the use of either Kraken2 or Centrifuge, and so these methods could be preferred for shorter reads if speed is a consideration: Kraken2 having the lower false negative rate, and Centrifuge the lower false positive rate (for longer reads, we found no discernible difference).

An additional benefit of Kraken2 and Centrifuge is that their databases can easily be customized to contain multiple human genomes rather than a singular reference (of which the current human reference is essentially an idiosyncratic type specimen [44]). This approach would expand the number of human k-mers (against which k-mers from the reads are compared) to include those from divergent regions, such as the HLA, and could also incorporate population-specific sequence (e.g. recent deep sequencing of 910 individuals of African descent identified 296 Mb of sequence missing from the GRCh38 reference [45]). Reads originating from population-specific regions would not be identifiable using either of the approaches tested in this study. An additional complication with alignment-based, relative to classification-based, methods is that as divergence increases relative to a reference the number of reads correctly mapped to the reference will decrease, as previously demonstrated for influenza A [46]. While in our simulations, viral reads (including from influenza A) could be consistently mapped to the same reference genome from which they were sequenced, we would not expect this to be as pronounced for non-simulated reads (i.e. where the genome from which the reads were derived diverges from the reference).

Alignment-based methods can, however, allow a finer degree of discrimination than classification-based methods. An alternative alignment-based approach would be to align reads (that are nominally from one bacterium) not just to the human genome but to the human and bacterial references simultaneously, as in the ‘remove contamination’ module of Clockwork (https://github.com/iqbal-lab-org/clockwork), a pipeline developed for the CRyPTIC project to process reads from *M. tuberculosis *. For reads with multiple mapping locations, we can conceivably have finer discrimination between human and non-human sequence. For instance, if a read, or one mate of a pair of reads, maps both to the human and microbial genomes, we could decide whether to consider those reads as human or microbial in origin, so maximizing either the number of human (or human-like) reads removed or the number of microbial (or microbial-like) reads retained.

In terms of memory requirements, while both classifiers drew upon reasonably large databases (∼8 Gb in each case), aligners also required a pre-built index of the (∼3 Gb) human genome as input. These indices varied 10-fold in size, from 2.3 Gb (SMALT) to 4.0 Gb (Bowtie2), 4.3 Gb (HISAT2), 5.1 Gb (BWA), 6.9 Gb (Minimap2), 8.1 Gb (Novoalign), 12.9 Gb (GEM) and 28.9 Gb (SNAP). One of the highest-performing methods in this study, Bowtie2 followed by SNAP, accordingly had among the largest runtime and memory requirements.

Nevertheless, it is important to take an exhaustive approach to depleting human reads from microbial read datasets. This is because as variant databases increase in scope and volume, it should become increasingly likely that personally identifiable phenotypic characteristics could be recovered from a small number of reads. This study has demonstrated that the subtractive alignment of human reads, if using only one aligner to do so, will probably be insufficient. Further to this, it remains a strong possibility that there is endemic, albeit low-level, human read contamination of (older) microbial read datasets in public archives.

## Methods

### Simulating sets of human-contaminated microbial reads

We obtained the NCBI reference genomes (criteria for which are detailed at https://www.ncbi.nlm.nih.gov/refseq/about/prokaryotes/, accessed 16 August 2018) for 10 clinically common species with fully sequenced (closed) core genomes, as detailed in Table S1, alongside versions GRCh38.p12 and GRCh37.p13 of the human primary assembly (i.e. the consensus genome excluding alternate haplotypes and patches; ftp://ftp.ensembl.org/pub/release-96/fasta/homo_sapiens/dna/Homo_sapiens.GRCh38.dna.primary_assembly.fa.gz and ftp://ftp.ensembl.org/pub/release-75/fasta/homo_sapiens/dna/Homo_sapiens.GRCh37.75.dna.primary_assembly.fa.gz, respectively; downloaded 13 April 2019).

From each bacterial genome, three sets of 150 and 300 bp paired-end short reads (characteristic of the Illumina NextSeq and MiSeq sequencers, respectively) were simulated using dwgsim v0.1.11 (https://github.com/nh13/DWGSIM) with parameters -e 0.001–0.01 (non-uniform per-base error rate increasing across the read from 0.01 to 0.1 %, approximating an Illumina error profile) and -y 0 (0 % probability of simulating a random DNA read). All reads were simulated with an insert size of ((read length × 2) + (read length × 0.2)), i.e. 150 bp reads were simulated with an insert size of 330 bp. dwgsim does not output the otherwise randomly generated seed used for each simulation, although does allow seeds to be provided. To ensure results were reproducible, the same seeds were used for each of the three replicates of each read length: 123 456 789, 234 567 891, and 345 678 912, respectively. The number of reads sequenced for each genome is equivalent to a mean base level coverage of 10-fold (for bacteria) or 100-fold (for viruses), as detailed in Table S2.

To each of these microbial read sets was added a number of human reads equivalent to between 1 and 10 % of the microbial reads, at increments of 1 %. All human reads were simulated from either GRCh38.p12 or GRCh37.p13 and used the same dwgsim seeds and parameters as above. Notably, dwgsim assigns read names on the basis of their origin. In this way, we can easily determine which of a set of reads predicted to be either human or microbial are correctly classified: true microbial reads will have names corresponding to microbial chromosome IDs.

### Methods for detecting human read content

Two basic methods were used for detecting human reads within the contaminated microbial read datasets: by alignment of all reads against the human genome, and by predicting read origin using the taxonomic classification tools Centrifuge v1.0.4 [19] and Kraken2 v2.0.7 [20]. Alignments were performed using BWA-mem v0.7.17 [25], Bowtie2 v2.3.4.1 [47], GEM v3.6.1–16 [26], HISAT2 v2.1.0 [27], minimap2 v2.10-r773 [28], Novoalign v3.09.00 (www.novocraft.com), SMALT v0.7.6 (http://www.sanger.ac.uk/science/tools/smalt-0) and SNAP v1.0beta.18 [29], in all cases with default parameters (except HISAT2, for which we set the -no-spliced-alignment parameter, required when giving DNA-seq reads as input but off by default). All alignments were made against two different builds of the human genome: the primary assembly of GRCh38.p12 (corresponding to GenBank assembly ID GCA_000001405.27 and obtained from Ensembl v96), and the primary assembly of GRCh37.p13 (corresponding to GenBank assembly ID GCA_000001405.14 and obtained from Ensembl v75).

In each case, BAM files were cleaned, sorted and duplicate-marked using Picard Tools v2.17.11 CleanSam, SortSam and MarkDuplicates, respectively [48]. Supplementary and non-primary alignments were removed using SAMtools view v1.7 [49] with parameters -F 2048 F 256. Finally, we obtained the set of reads mapped by each aligner (i.e. considered human) using SAMtools view with parameters -F 4 f 8, -F 8 f 4, and -F 12, merging the resulting BAMs. This was in order to obtain reads where both ends mapped as well as the set of mapped reads with an unmapped mate, as the latter, being from the same DNA fragment, should also be of human origin. Read IDs were extracted from each BAM using the ‘bamToBed’ module of BEDtools v2.19.1 [50].

By contrast, the classification tools Centrifuge and Kraken2 compare sets of reads to a database of genomes, probabilistically assigning a taxonomic rank to each read. For both tools, we used one database containing only the human (GRCh38) genome and one containing the set of RefSeq bacterial, archaeal and viral genomes, plus the human genome. The human-only databases were custom-built using the same primary assembly from which human reads were simulated. For the larger multi-species databases, we obtained the pre-built Centrifuge ‘P+H+V’ database (ftp://ftp.ccb.jhu.edu/pub/infphilo/centrifuge/data/p+h+v.tar.gz, created June 2016) and the comparable MiniKraken (ftp://ftp.ccb.jhu.edu/pub/data/kraken2_dbs/minikraken2_v2_8GB_201904_UPDATE.tgz, created April 2019), both of which draw upon genomes stored in RefSeq.

### Human read detection in real sequencing data

To test for the presence of human reads in real bacterial sequencing data, we downloaded the daily-updated SRA BioProject summary file (*n*=383590 BioProjects; ftp://ftp.ncbi.nlm.nih.gov/bioproject/summary.txt, accessed 24 September 2019) and parsed it to extract a list of BioProject IDs with a data type of ‘genome sequencing’ and a species name matching, at least in part, the name of any of the 10 clinically common bacteria detailed in Table S1 (e.g. ‘* Salmonella enterica* subsp. *enterica* serovar *Oranienburg* str. 0250’ matches ‘* Salmonella enterica *’). We then used the Entrez Direct suite of utilities (https://www.ncbi.nlm.nih.gov/books/NBK179288/, accessed 1 May 2019) to associate each BioProject ID with a list of SRA sample and run IDs (a ‘RunInfo’ file). RunInfo files were parsed to retain only those runs where ‘Platform’ was ‘ILLUMINA’, ‘Model’ (i.e. sequencer) was ‘HiSeq 2000’, ‘HiSeq 2500’ or ‘MiSeq’ (all of which use TruSeq3 reagent chemistry), ‘LibrarySource’ was ‘GENOMIC’ or ‘METAGENOMIC’, ‘LibraryStrategy’ was ‘WGS’, ‘LibraryLayout’ was ‘PAIRED’, ‘LibrarySelection’ was ‘RANDOM’, ‘avgLength’ was ≥150 (i.e. mean read length of 150 bp), and ‘spots’ was >1 and <5 (i.e. approximating a read depth of >1 and <5 million reads, the upper limit chosen to minimize the computational cost of data processing). This generated a list of 11 577 SRA sample IDs, representing sequencing data from 356 different BioProjects.

From each sample, human reads were classified using the two-stage approach of Bowtie2 followed by SNAP (detailed above). These reads were extracted from the original fastq files using the ‘subseq’ module of seqtk v1.3 (https://github.com/lh3/seqtk) and aligned to the human genome (GRCh38.p12) using BWA-mem v0.7.17 with default parameters (BWA was chosen as it was among the most sensitive aligners tested). The resulting BAMs were post-processed using Picard Tools to clean and duplicate-mark (as above), with the subset of mapped reads obtained using SAMtools view v1.7 with parameter -F 4. SNPs were called using BCFtools mpileup [49] with parameters -A (do not discard anomalous read pairs, i.e. include singleton ‘orphan’ reads) and -B (disable probabilistic realignment for the computation of base alignment quality scores). These parameters were chosen to maximize the likelihood of SNP calling at extremely low depth, with the intention that lower-confidence calls could later be identified (see below).

The resulting VCF was annotated using BCFtools ‘annotate’ to assign dbSNP IDs, where available, to those positions already known to be *bona fide* human SNPs. For this purpose, we obtained two sets of known human SNPs: those with clinical assertions, which are included in ClinVar (ftp://ftp.ncbi.nlm.nih.gov/pub/clinvar/vcf_GRCh38/clinvar_20191013.vcf.gz, accessed 21 October 2019), and the set of dbSNP ‘common SNPs’ (ftp://ftp.ncbi.nih.gov/snp/organisms/human_9606/VCF/common_all_20180418.vcf.gz, accessed 21 October 2019). ‘Common’ SNPs are defined as having both a germline origin and a minor allele frequency of ≥0.01 in at least one of 26 major human populations (according to the 1000 Genomes Project [51]; https://www.internationalgenome.org/category/population), with at least two unrelated individuals having the minor allele (https://www.ncbi.nlm.nih.gov/variation/docs/human_variation_vcf/, accessed 21 October 2019).

Finally, higher-confidence human SNPs were considered those which had (a) ≥2 reads supporting the alternative allele [these counts were obtained from the DP4 field of the mpileup VCF, and automatically exclude low-quality bases (by default, those with Phred <13)], (b) QUAL value ≥20, (c) MAPQ value of 60 (used by BWA to indicate unique mapping) and (d) could be assigned a ‘common’ dbSNP ID.

### Evaluation metrics

For each method of human read detection, we obtained the number of true positive (TP; human read classified as human), false positive (FP; bacterial read classified as human) and false negative (FN; human read not classified as human) read classifications. We then calculated the precision (positive predictive value) of each method as TP/(TP+FP), recall (sensitivity) as TP/(TP+FN), and as an ‘overall performance’ measure, the *F*-score (as in [30]), which equally weights precision and recall: *F*=2 × ((precision × recall)/(precision +recall)). The *F*-score summarises the performance of each method as a value bound between 0 and 1 (perfect precision and recall).

### Gene ontology (GO) term enrichment

GO term enrichment was assessed using the R/Bioconductor package topGO v2.36.0, which implements the ‘weight’ algorithm to account for the nested structure of the GO tree [52]. This is a closed testing procedure – the computation of *P* values per GO term is conditional on the neighbouring terms, and so the tests are not independent. For this reason, *P* values produced by the ‘weight’ algorithm are interpreted as corrected or not affected by multiple testing. topGO requires a reference set of GO terms as input. These were built from the GRCh38.p12 and GRCh37.p13 sets (obtained via Ensembl BioMart versions 96 and 75, respectively), each filtered to remove terms with evidence codes ‘NAS’ (non-traceable author statement) and ‘ND’ (no biological data available), and those assigned to fewer than 10 genes in total. Significantly enriched GO terms (*P* <0.05) were retained only if the observed number in each set of genes exceeded the expected number by 2-fold or greater.

## Data Bibliography

1. Public sequencing data for 10 bacterial species: 356 NCBI BioProject accessions provided in Table S8 (data for each BioProject is accessible via the URL template https://www.ncbi.nlm.nih.gov/bioproject/X, where X is a BioProject accession).

## Supplementary Data

Supplementary material 1

Supplementary material 2
