# Supplementary material for: Evaluation of methods for detecting human reads in microbial sequencing datasets
Source: Microb Genom. 2020 Jun 19;6(7):mgen000393. doi: 10.1099/mgen.0.000393 (PMC7478626; doi:10.1099/mgen.0.000393)
Supplement: Supplementary material 1 [file mgen-6-393-s001.pdf]

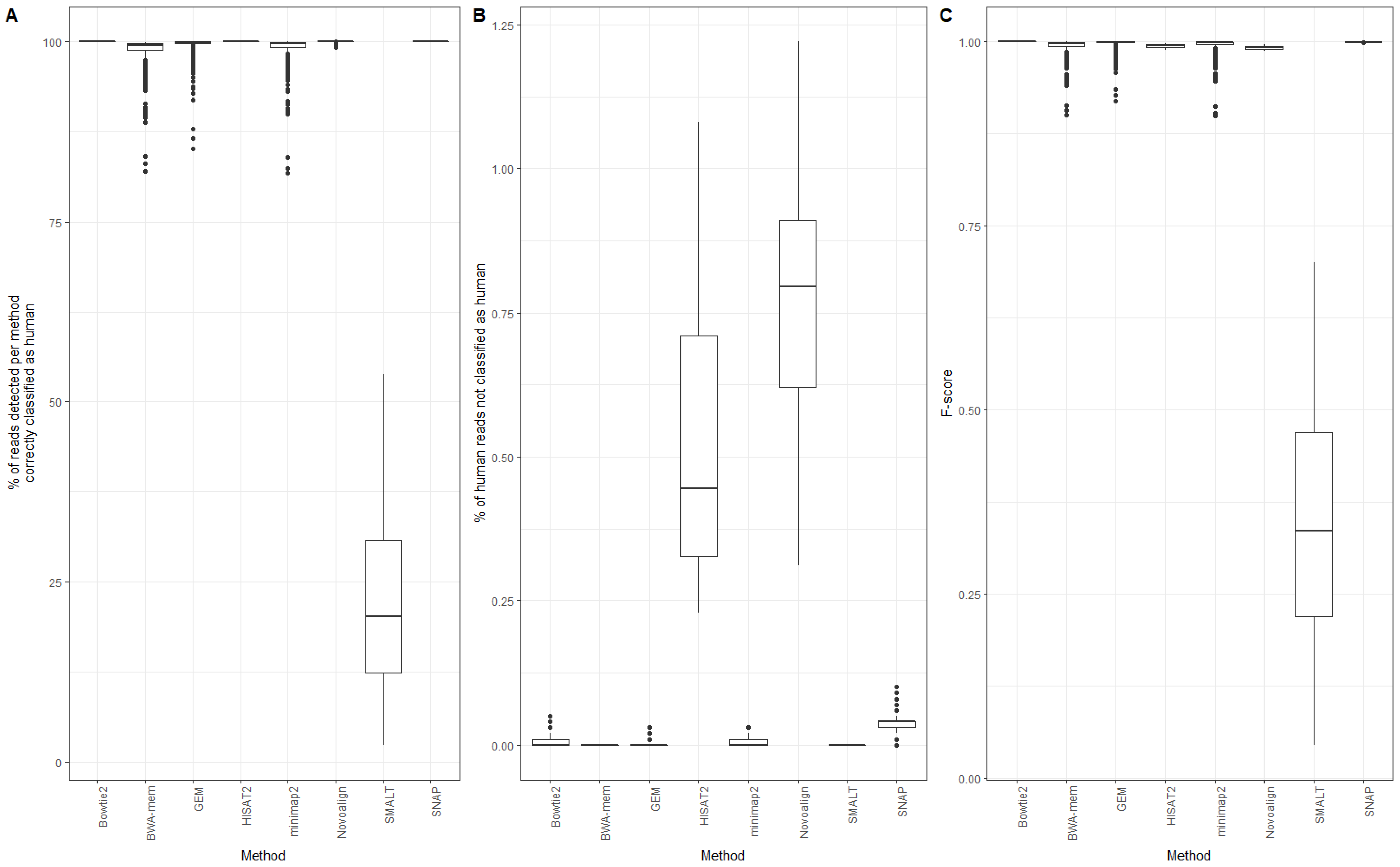
A
% of reads detected per method correctly classified as human
100
75
50
25
0
Bowtie2
BWA-mem
GEM
HISAT2
minimap2
Novoalign
SMALT
SNAP
Method
B
% of human reads not classified as human
1.25
1.00
0.75
0.50
0.25
0.00
Bowtie2
BWA-mem
GEM
HISAT2
minimap2
Novoalign
SMALT
SNAP
Method
C
F-score
1.00
0.75
0.50
0.25
0.00
Bowtie2
BWA-mem
GEM
HISAT2
minimap2
Novoalign
SMALT
SNAP
Method
